# It is time for home-based 12-lead electrocardiogram

**DOI:** 10.1093/europace/euac138

**Published:** 2022-08-25

**Authors:** Ruey-Kang Chang

**Affiliations:** Harbor-UCLA Medical Center, Torrance, CA, USA

In the editorial ‘Is it time for a consumerized or home-based 12-lead electrocardiogram?’, Tooley and Turakhia^1^ pointed out the important limitations of one-lead electrocardiogram (ECG) or multi-lead ECG without precordial leads. They called for home based 12-lead ECG because ‘The hospital 12-lead ECG remains too cumbersome, and adapting a single left arm-right arm smartwatch ECG to obtain additional vectors is not ideal due to the high potential for baseline artefact and user error.’

I agree with the authors that ‘We need “quick and easy” 12-lead ECG systems—not just for the consumer, but for a pre-hospital, emergency, inpatient, and outpatient care.’ In a study of 12-lead ECG performed at home by patients with no training or experience, it was shown that over 92% patients completed their tests successfully, and technical failure was <2%.^2^

I believe 12-lead ECG will soon join blood pressure, pulse oximetry, glucose, and one-lead ECG to be a test that we offer to patients to do at home. Home-based 12-lead ECG will greatly enhance the capability of screening diseases in silent state, early detection of subtle clinical changes, and follow up of patients with symptomatic diseases.
